# The genus *Diolcogaster* Ashmead, 1900 (Hymenoptera, Braconidae, Microgastrinae) from China

**DOI:** 10.3897/zookeys.129.1201

**Published:** 2011-09-16

**Authors:** Jie Zeng, Jun-hua He, Xue-xin Chen

**Affiliations:** State Key Lab for Rice Biology, Institute of Insect Sciences, Zhejiang University, 268 Kaixuan Road, Hangzhou 310029, China

**Keywords:** Hymenoptera, Braconidae, Microgastrinae, *Diolcogaster*, new species, China, key

## Abstract

The genus *Diolcogaster* Ashmead, 1900 (Hymenoptera, Braconidae, Microgastrinae) from China is revised and keyed, with nine new species, namely *Diolcogaster bifurcifossa* **sp. n.**, *Diolcogaster brevivena* **sp. n.**, *Diolcogaster grammata* **sp. n.**, *Diolcogaster ineminens* **sp. n.**, *Diolcogaster laetimedia* **sp. n.**, *Diolcogaster pluriminitida* **sp. n.**, *Diolcogaster praritas* **sp. n.**, *Diolcogaster punctatiscutum* **sp. n.** and *Diolcogaster translucida* **sp. n.** described and illustrated, and one species, *Diolcogaster perniciosa* (Wilkinson, 1929) recorded for the first time from China. A key to the Chinese species of this genus is provided.

## Introduction

The genus *Diolcogaster* (Braconidae, Microgastrinae) was proposed by Ashmead (1900) based on the type species, *Microgaster melligaster* Provancher, 1886, that was later transferred into the genus *Hygroplitis* Thomson, 1895 (Mason 1981). Mason (1981) placed the bulk of those species grouped by Nixon (1965) under *Protomicroplitis* Ashmead, 1898 in this genus, excluding the species of the *calceata*-, *marginata*-, *lepelleyi*-, *calliptera*-, *schunkei*- group and some New World species not known to Nixon, and synonymized *Zadiolcogaster* with *Diolcogaster*. Saeed et al. (1999) revised the Australasian *Diolcogster*, and described more than 20 species.

At present, this genus includes 66 described species widespread in the world (Ashmead 1900; Viereck 1913; Muesebeck 1922; Wilkinson 1929, 1930; Granger 1949; Muesebeck and Walkley 1951; Telenga 1955; Nixon 1965; Tobias 1971, 1976; You et al. 1990; Saeed et al. 1999; Luo and You 2003, 2005; Chen and Song 2004; Yu et al. 2005), of which only four species, *Diolcogaster alvearia* (Fabricius, 1798), *Diolcogaster facetosa* (Weed, 1888), *Diolcogaster chaoi* (Luo & You) and *Diolcogaster spreta* (Marshall, 1885), were once reported to occur in China (Fahringer 1935; You et al. 1990; Luo and You 2003, 2005; Chen and Song 2004). However, the Nearctic species *Diolcogaster facetosa* was obviously misidentified by Chen and Song (2004) in China because Nixon (1965) stated that this species has the ovipositor sheath with 2–3 blackened setae at its apex and apical segment of front tarsus with a less well developed lateral spine while Chen and Song (2004) described this species with the ovipositor sheath only with setae at apex, and apical segment of front tarsus without a lateral spine.

Species of this genus are parasitoids of Lepidoptera hosts that include the family Arctiidae, Geometridae, Lasiocampidae, Limacodidae, Lymantriidae, Noctuidae, Notodontidae, Plutellidae, Pyralidae, Tenthredinidae, and Thaumetopoeidae (Marshall 1885; Muesebeck 1922; Wilkinson 1929; Granger 1949; Hedwig 1950; Telenga 1955; Nixon 1965; Tobias 1971, 1976; Saeed et al. 1999), some of which are important pests causing damages on agricultural production.

Recently nine new species and a newly recorded species, *Diolcogaster perniciosa* (Wilkinson, 1929) of this genus were founded from China when we examined specimens of Parasitic Hymenoptera Collection of Zhejiang University (ZJUH) during an on-going project on the revision of the Chinese Microgastrinae.

## Material and methods

Specimens studied are deposited in the Parasitic Hymenoptera Collection of Zhejiang University, Hangzhou, China (ZJUH). Descriptions and measurements were made under a stereomicroscope (Zeiss Stemi 2000-C). All photographs were made with a Leica DFC425 Camera attached to a stereomicroscope (Leica M205 A, Germany) and Leica Application Suite version 3.60 software.

Terminology and measurement follows Nixon (1965) and Mason (1981), veins follows the modified Comstock-Needham system (van Achterberg 1979). Abbreviations used in this paper are as follows: POL = postocellar line, OOL = ocular-ocellar line, OD = ocellar diameter; T1 = the 1st tergite of metasoma, T2 = the 2nd tergite of metasoma, T3 = the 3rd tergite of metasoma; L = length, W = width.

## Taxonomy

### Genus Diolcogaster

Ashmead, 1900

http://species-id.net/wiki/Diolcogaster

Diolcogaster Ashmead 1900, 23(1206): 132; Viereck 1913, 46: 366; Viereck 1914, 83: 46; Viereck (1916)1917, 22: 202; Lyle 1918 51: 104, synon. by Szépligeti; Mason 1981 115: 113; Marsh et al. 1987 13: 32; Austin and Dangerfield 1992, 6(1): 5, 25; Whitfield 1997, No. 1: 337; Chen and Song 2004: 175. Type species: *Microgaster brevicaudus* Provancher, 1886. Designated by Viereck 1914.
Zadiolcogaster Viereck 1913, 46: 366; Viereck 1921, 59: 147, synon. by Muesebeck and Walkley 1951.Type species: *Zadiolcogaster anomus* Viereck, 1913.

#### Diagnosis.

 Areolet of fore wing present, large or small, quadrangular or triangular; vannal lobe with margin usually convex and setose but sometimes varying to straight and setose and exceptionally concave and without setae. Side of pronotum always with ventral groove, rarely with dorsal groove. Propodeum usually rugose but occasionally smooth; median longitudinal carina strong and complete.

First tergite of metasoma bearing a sharp median longitudinal groove through most of its length; but varying from short and expanded apically to strongly narrowed, lorate. Second tergite with well- or ill-defined median field. Third tergite smooth, but strongly rugose when the second one is similarly sculptured. Hind coxa large, inner hind tibial spurs usually long.

Hypopygium short and inflexible. Ovipositor sheaths short, usually with dense setae near the apex; sheaths of most species with a few modified strong setae.

#### Key to species of the genus Diolcogaster Ashmead, 1900 from China

**Table:** 

1	Tip of scutellum polished, not interrupting posterior, polished band of scutellum at middle; vein r-m of fore wing arising from vein r+3-SR so that areolet is 4-sided	**2**
–	Rugose tip of scutellum interrupting posterior, polished band of scutellum at middle; areolet of fore wing various in shape	**5**
2	Metacarp short, shorter than twice as long as its distance from apex of marginal cell; T2 highly polished, shorter than T3	*Diolcogaster brevivena* **Zeng & Chen, sp. n.**
–	Metacarp longer, at least twice as long as its distance from apex of marginal cell; T2 more or less rugose to rugulose, slightly longer than T3	**3**
3	Propodeum with a weak and incomplete median keel	*Diolcogaster alvearia* **(Fabricius, 1798)**
–	Propodeum with a strong and complete median keel	**4**
4	Vein r of fore wing as long as or slightly shorter than vein 2-SR; disc of scutellum finely, discretely punctate; T2 with a shiny and feebly longitudinally striated raised median field, irregularly shaped but distinctly narrowed behind; the lateral fields striate-rugose, but reduced toward lateral margin	*Diolcogaster praritas* **Zeng & Chen, sp. n.**
–	Vein r of fore wing much longer than vein 2-SR; disc of scutellum with dense punctures, larger laterally; T2 transverse, shortened medially, with a raised pentagonal median field, shiny but densely rugulose all over	*Diolcogaster punctatiscutum* **Zeng & Chen, sp. n.**
5	Antennae of female very short, segments 10–12 being hardly longer than wide; metacarp short, shorter than 1.5× as long as its distance from apex of marginal cell; T2 divide by two curved, more or less rugose and ][-shaped grooves into three fields of which the middle one is triangular and narrowest behind	*Diolcogaster spreta* **Marshall,1885**
–	Antennae of female long, all segments distinctly longer than wide; metacarp longer, at least twice as long as its distance from apex of marginal cell; T2 never that sculptured	**6**
6	Vein r-m of fore wing arising from vein r+3-SR so that the areolet is 4-sided; vein 1-CU1 much shorter than 2-CU1	**7**
–	Vein r-m of fore wing more or less interstitial with vein r (1st abscissa of the radius) so that the areolet is virtually 3-sided; relative length of vein 1-CU1 variable	**8**
7	T2 as long as T3; T3 with traces of rugosity; vein r arising from distal 1/2 of pterostigma, far behind the middle	*Diolcogaster laetimedia* **Zeng & Chen, sp. n.**
–	T2 distinctly shorter than T3; T3 highly polished; vein r arising from distal 1/2 of pterostigma, but only slightly behind the middle	*Diolcogaster perniciosa* **(Wilkinson, 1929)**
8	T3 as heavily sclerotised as T2, the two tergites together forming a sculptured carapace, beneath which the more apical tergites are completely or partially hidden; fore wing marked with brown patches at apex	**9**
–	T3 usually smooth, polished and never forming a carapace with T2 such as above; fore wing without patches at apex	**10**
9	Ovipositor sheath with a thickened seta; T1 distinctly widened posteriorly; posterior margin of T3 rounded, emarginated medially; forewing with apical spot dark brown	*Diolcogaster chaoi* **(Luo & You, 2003)**
–	Ovipositor sheath without trace of apical, modified setae; T1 slightly widened posteriorly; T3 subrectangular, with posterior margin straight, not emarginated medially; forewing with apical spot slightly infuscate	*Diolcogaster bifurcifossa* **Zeng & Chen, sp. n.**
10	T2 without trace of median field, rugose more or less all over; vein 1-CU1 distinctly shorter than 2-CU2; mesoscutum shiny, with very dense and evenly discrete punctures	*Diolcogaster ineminens* **Zeng & Chen, sp. n.**
–	T2 with a more or less distinct median field that varies in shape but is never triangular or widened posteriorly, though sometimes is widened anteriorly; vein 1-CU1 slightly longer than 2-CU2; sculptures of mesoscutum variable	**11**
11	T1 linear, very long and narrow, more than thrice as long as its width; propodeum with weak median keel; T2 with a distinctly raised pentagonal median field that is sharply-sided and with smooth and shiny surface, area beside median field setose and also smooth and shiny; pterostigma brown, except basal pale spot	*Diolcogaster grammata* **Zeng & Chen, sp. n.**
–	T1 not linear, not more than 2.5× as long as its width; propodeum with strong complete median keel; T2 without such a raised pentagonal median field; pterostigma without a basal pale spot	**12**
12	T2 with an elongated median field that is not triangularly widened behind; vein 1-R1 more than four times as long as its distance from the apex of the marginal cell; POL much longer than OD; vertex and frons densely rugose; face at upper half with very indistinct median longitudinal carina	*Diolcogaster translucida* **Zeng & Chen, sp. n.**
–	T2 with an ill-defined median field, only slightly swollen; vein 1-R1 with distal end almost reaching to apex of marginal cell; POL about 1.5× as long as OD. Vertex finely transversely striate; frons polished and without setae in large part but densely rugulose and setose adjacent to eye margin; face without any trace of median keel	*Diolcogaster pluriminitida* **Zeng & Chen, sp. n.**

### Diolcogaster alvearia

(Fabricius, 1798)

http://species-id.net/wiki/Diolcogaster_alvearia

Ichneumon alevarius Fabricius 1798: 232. Holotype female, pinned with labels as follows: “Galliae.” (Kiel)-Zimson 1964 Type Material Fabricius: 369.
Cryptus alvearius Fabricius 1804, 2: 90.
Microgaster alvearius (*-ia*): Spinola 1808: 149; Curtis 1830, 6: 321; Haliday 1834: 240; Nees von Esenbeck 1834: 174; Ratzeburg 1852: 50; Ruthe 1860, 4: 153; Fitch 1883, 16: 167; Marshall 1885: 240; Marshall 1890: 516; Lyle 1918: 107; Fahringer 1935, 27A(12)(1934): 6; Fahringer 1937, 4(4–6): 331; Telenga 1955, 5(4): 184; Papp 1959, 51:399; Papp 1960, 12: 119.
Protomicroplitis alevarius :Nixon 1965: 250.
Diolcogaster alvearia : Mason 1981: 114; Chen and Song 2004: 175.

#### Host.

*Alcis repandata*; *Croesus septentrionalis*; *Hypomecis* [*Clematis*]; *Menophra abruptaria*; *Opisthograptis luteolata*; *Ourapteryx sambucaria*; *Peribatodes rhomboidaria* (Yu & van Achterberg, 2004).

#### Distribution.

 China (Gansu, Fujian); Austria, Bulgaria, Czechoslovakia, France, Germany, Hungary, Italy, Moldova, Netherlands, Romania, Russia, Slovakia, Slovenia, Spain, Switzerland, Turkey, United Kingdom, Yugoslavia.

#### Remarks.

 No specimens were available for this study. However, the description of this species by Chen and Song (2004) based on the specimens collected from Fujian is obviously different from that by Telenga (1955). Telenga described this species with mesoscutum coriaceous-rugose, dull; areolet 4-sided; and T1 parallel-sided while Chen & Song stated that this species has the mesoscutum with fine punctures anteriorly but almost smooth posteriorly; areolet 3-sided; and T1 broadening posteriorly. Therefore, the specimens identified by Chen & Song (2004) need further examination.

### Diolcogaster bifurcifossa

Zeng & Chen sp. n.

urn:lsid:zoobank.org:act:C726589D-4248-47EA-A0C1-BCC08D81B09D

http://species-id.net/wiki/Diolcogaster_bifurcifossa

Figs 1–8

#### Description.

 Female. Body length 3.4 mm, fore wing length 3.8 mm.

Head. Rather large, oval in frontal view, with antennal sockets high above middle level of eyes; transverse in dorsal view, 1.6× as wide as long and as wide as mesoscutum, with short white setae all over, including eyes. Ocelli in a very low triangle, the posterior transverse tangent to the anterior ocellus cutting the posterior pair. POL:OD:OOL=7.1:5.0:6.0. Vertex shiny but densely and shallowly punctate, strongly convex, and sharply constricted to occiput; frons virtually without sculpture; face shiny but shallowly rugose, slightly convex, upper half with indistinct median longitudinal carina which is triangularly widened downwards and forms a subtriangular shiny area; width of face 0.6× as high as eye and clypeus combined (19.8:32.2); eyes very large, inner margin of eyes slightly constricted at antennal sockets, widening upwards and downwards, 1.7× as high as wide (30.0:17.9); temple and gena also shiny but shallowly rugose, densely setose. Mouth opening wide, clypeus feebly rugose; tentorial pits of moderate size, distance between tentorial pits 3.6× as long as distance from pit to eye margin (11.0:3.1); malar space very short, 0.2× as long as eye height. Antennae normal, long, with the preapical segment about 2.3× as long as wide; flagellomeres long, thick, with bristly setosity, all flagellomeres with placodes arranged regularly in 2 ranks; apical segment longer than preapical one. Flagellomere proportion: 2 L/W=2.6, 8 L/W=2.6, 14 L/W=2.2; L 2/14=1.4; W 2/14=1.2.

Mesosoma. Pronotum shiny but sparsely punctate, with a strongly indicated, foveate, ventral furrow laterally. Mesoscutum shiny, evenly and densely punctate, with sparse short setae all over; notauli not impressed. Disc of scutellum shiny, also evenly punctate, its rugose (punctate) tip at middle widely interrupting the posterior, polished band of scutellum, with sparse short setae. Lateral part of the polished band at least distinctly convex anteriorly. Anterior margin of the postscutellum closely applied to the posterior margin of the scutellum so that, laterally, the phragma of scutellum is completely hidden. Scutellar sulcus deep, with few strong longitudinal short carinae, its width 1.3× as long as scutellum (25.0:20.0). Propodeum short, with a strong median keel, shiny beside the median keel on posterior half and anteriorly just behind anterior margin; the area behind the anterior polished area and posterolaterally strongly setigerous punctate; spiracles enclosed by strong costulae. Mesopleuron polished posteriorly and above precoxal sulcus polished; precoxal sulcus long, broad, shallow, punctate.

Wings. Forewing with vein r-m reduced to a hyaline point; areolet 3-sided; vein r (1st abscissa of radius) arising from distal 1/2 of pterostigma, far behind the middle, very obliquely placed on the pterostigma; meeting vein 2-SR at a 150 degree angle. Vein 1-R1 (metacarp) with distal end sharply defined, 5.5× as long as its distance from the apex of the marginal cell and 1.1× as long as pterostigma; pterostigma 3.3× as long as wide; r:2-SR:length of pterostigma=14.5:6.0:40.0. Width of 1st discal cell:height of 1st discal cell =31.5:38.0; 1-CU1:2-CU1:m-cu=13.0:12.5:12.5. Hind wing narrow, with edge of vannal lobe beyond its widest part very slightly concave and without trace of a fringe of setae.

Legs. Long. Hind coxa large, flattened on outer side, closely covered with deep punctures, the interspaces very shiny, hardly reaching past posterior margin of T3. Hind tibia swollen toward apex and 0.9× as long as hind tarsus (70.0:80.0). Inner hind tibial spur much longer than outer one, about 0.7× as long as hind basitarsus (23.0:33.0); fourth tarsal segment shorter than fifth tarsal segment (9.0:12.0); apical segment of the front tarsus without a spine. Tarsal claws long, simple.

Metasoma. Shorter than mesosoma. T1 strongly sclerotised, slightly widened posteriorly, with a complete deep and broad median groove which bifurcates apically into two strongly foveate grooves, with smooth surface but setigerous punctate distal to the middle, 1.4× as long as its width and 1.2× as long as T2. T2 with a polished elongated, narrow, parallel-sided median field that is separated from the strongly striate-rugose lateral surface by two broad, deeply foveate grooves; 0. 7× as long as its greatest width and 1.2× as long as T3. T3 as heavily sclerotised as T2, the two tergites together forming a sculptured carapace, beneath which the more apical tergites are completely or partially hidden. T3 subrectangular, with middle of anterior margin slightly arched; very shiny, with surface aciculate, coarsely laterally and weakly medially; the weak but distinct polished median field slightly widened posteriorly and open apically; separated from T2 by a deep foveate groove that is margined along posterior edge of T2. Tergites posterior to T3 membranous, smooth and shiny with very few fine setae. Ovipositor sheath thin and decurved, with sparse long setae posteriorly, without trace of apical, modified setae. Hypopygium large, evenly sclerotised, smooth and without setae, slightly surpassing the last tergite.

Colour. Body bright yellow to black; head brown, except the yellow mouthparts; mesosoma black, but brownish. Flagellum yellowish brown at basal half and darkened to brown apically, scape and pedicel bright yellow, even with antennal socket lightened to yellowish apically. Apex of mandible brown; palpi whitish yellow. Tegula yellow. Fore and mid legs bright yellow. Hind coxa, trochanter and femur also bright yellowish, except the extreme apex of coxa and distal 1/4 of femur brown; hind tibia and tarsi brown except the area distal to the extreme base to the middle of the tibia, the base of basitarsus and the tibial spurs whitish yellow. T1 bright yellow, somewhat transparent, T2 and T3 black, the other tergites and ovipositor sheaths light brown, with posterior margin of tergites more or less transparent, whitish. Wings hyaline but forewing with a faint apical spot; veins brown, vein 1-R1 (metacarp) lightened to yellow, pterostigma evenly brown with extreme base yellowish.

Variation. Body length 2.7–4.2mm, sometimes few tergites posterior to T3 with pale colour. Some individuals from Hainan with pale-colored head and some other individuals with T1 darkened posteriorly.

**Figures 1–8.**
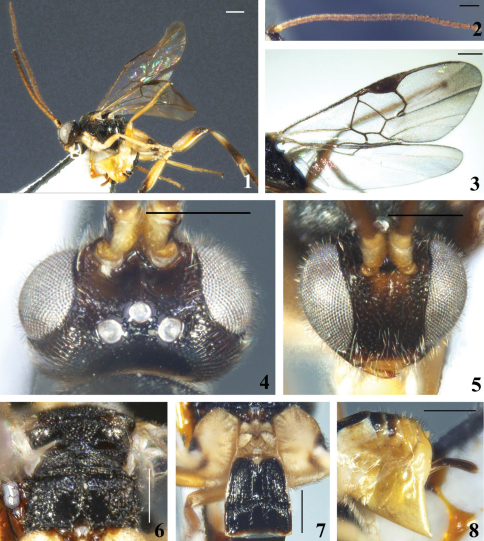
*Diolcogaster bifurcifossa* Zeng & Chen, sp. n. **1** habitus, lateral view **2** antennae **3** fore wing **4** head, dorsal view **5** head, frontal view **6** scutellum and propodeum, dorsal view **7** T1–3, dorsal view **8** hypopygium and ovipositor sheaths, lateral view. Scale line = 0.5 mm.

#### Male.

 Same as female.

#### Host.

 Unknown.

#### Material examined.

 Holotype: ♀, Mt. Diaoluo (109°53'E, 18°39'N ~ 109°58'E, 18°42'N), Lingshui, Hainan, 2006. VII. 16–17, Liu Jingxian, No. 200802405. Paratype: 1♀, Mt. Diaoluo (109°53'E, 18°39'N ~ 109°58'E, 18°42'N), Lingshui, Hainan, 2006. VII. 16–17, Weng Liqiong, No. 200802525; 4♂♂, Mt. Diaoluo (109°53'E, 18°39'N ~ 109°58'E, 18°42'N), Lingshui, Hainan, 2006. VII. 16–17, Liu Jingxian, No. 200802161, 200802163, 200802289, 200802419; 1♀, Mt. Jianfengling (108°48'E, 18°41'N ~ 108°49'E, 18°42'N), Ledong, Hainan, 2007. VI. 5–7, Xiao Bin, No. 200806874; 2♀♀, Tianchi, Mt. Jianfengling (108°48'E, 18°41'N ~ 108°49'E, 18°42'N), Ledong, Hainan, 2007. X. 22–23, Liu Jingxian, No. 200810501, 200810789; 1♀, Hongmao, Mt. Yinggeling (109°31'E, N19°04'N), Baisha, Hainan, 2007. V. 23–25, Zeng Jie, No. 200804476; 1♀1♂, Mt. Yinggeling (109°31'E, N19°04'N), Baisha, Hainan, 2007. X. 18, Liu Jingxian, No. 200709927, 200709723; 1♀, Mt. Yinggeling (109°11'E, 18°49'N ~ 109°34'E, 19°08'N), Baisha, Hainan, 2008. XI. 17, Wang Manman, No. 200805450; 1♂, Mt. Bawangling (109°02'E, 19°05'N ~ 109°04'E, 19°08'N), Changjiang, Hainan, 2008. XI. 26, Wang Manman, No. 200805645; 1♀, Mt. Chebaling (114°14'E, 24°43'N ~ 114°16'E, 24°44'N), Shixing, Guangdong, 2003. VIII. 21, Xu Zaifu, No. 20052280; 1♀, Mt. Nanling (112°59'E, 24°53'N ~ 113°05'E, 24°56'N), Ruyuan, Guangdong, 2004. VIII. 4, Xu Zaifu, No. 20049754; 1♀, Mt. Jiuwandashan (108°12'E, 24°52'N ~ 108°32'E, 25°03'N), Pingying, Guangxi, 2003. VII. 30, Wang Yiping, No. 20037539; 1♀, Shiwandashan Forest Park (107°53'E, 21°53'N ~ 107°55'E, 24°55'N, 310m), Guangxi, 2001. XI. 29, Ma Yun, No. 20021577; 1♀, Letu (117°13'E, 24°53'N ~ 117°14'E, 24°54'N), Nanjing, Fujian, 1991. V. 23, Liu Changming, No. 20006068; 1♀, Xianrending, West Mt. Tianmu (119°23'E, 30°20'N ~ 119°24'E, 30°20'N), Linan, Zhejiang, 1999. VI. 30, Zhao Mingshui, No. 996522; 2♀♀, Mt. Gutian (118°07'E, 29°14'N ~ 118°10'E, 29°16'N), Kaihua, Zhejiang, 2005. VII. 3, Zhang Hongying, No. 200616206, 200617220.

#### Etymology.

 The specific name “*bifurcifossa*” derives from the Latin adjective “bifurcus” and noun “fossa”, referring to median groove of T1 bifurcate apically.

#### Distribution.

 China (Zhejiang, Fujian, Guangdong, Guangxi, Hainan).

#### Remarks.

 This species is similar to *Diolcogaster eclectes* (Nixon, 1965), but can be distinguished by the almost smooth surface of T3 beside the median field (the latter has T3 sculptured except for a median, polished area) and the ovipositor sheath without a modified apical seta (the latter with one).

### Diolcogaster brevivena

Zeng & Chen sp. n.

urn:lsid:zoobank.org:act:86E56FF6-ECA7-421E-93E4-FBF455E82352

http://species-id.net/wiki/Diolcogaster_brevivena

Figs 9–16

#### Description.

 Female. Body length 2.7mm, fore wing length 2.8mm.

Head. Small, subquadrate in anterior view, with antennal sockets high above middle level of eyes; strongly transverse in dorsal view, 1.6× as wide as long and almost as long as mesoscutum, with very short fine setae except the sharply constricted and highly polished area behind lateral ocelli. Ocelli small, in a low triangle, but the transverse, posterior tangent to the anterior ocellus not cutting the posterior pair. POL:OD:OOL=4.0:3.0:7.0. Vertex vey shiny, flattened; frons polished, virtually without sculpture; face convex, also very shiny, with sparse fine punctures, feebly transverse striate on upper half, without any trace of median keel, width of face 0.8× as high as eye and clypeus combined (16.6:20.0); eyes small, inner margin of eyes parallel, 1.6× as high as wide (18.0:11.0); temple and gena shiny, densely and finely striate, with appressed longer setae. Clypeus separated from face by a fine curved line linking the tentorial pits, slightly convex and as finely and sparsely punctate as face; tentorial pits small, distance between tentorial pits 2.7× as long as distance from pit to eye margin; malar space short, 0.3× as long as eye height. Antennae much shorter than body, with the preapical segment only 1.2× as long as wide; flagellomeres slightly thickened apically, with bristly setosity, with placodes arranged regularly in 2 ranks; apical segment longer than preapical one. Flagellomere proportion: 2 L/W=3.0 , 8 L/W=1.5, 14 L/W=1.4; L 2/14=1.8; W 2/14=0.6.

Mesosoma. Pronotum shiny, with a broad ventral furrow laterally. Mesoscutum very shiny, very densely but finely punctate, with posterior margin distinctly rimmed, with dense short fine setae all over; notauli not impressed, only slightly depressed on the posterior imaginary course. Disc of scutellum polished, shiny and at most weakly punctate, with sparse fine setae; polished at tip, so the posterior, polished band of scutellum is continuous. Lateral part of the polished band distinctly convex anteriorly. Anterior margin of the postscutellum closely applied to the posterior margin of the scutellum so that, laterally, the phragma of scutellum is completely hidden. Scutellar sulcus shallow, with few indistinct longitudinal carinae, its width as long as scutellum (13.0:13.0). Propodeum short, shiny, with a strong complete median keel, smooth with very sparse fine punctures, except for very short transverse carina each side along the median keel and dense rugulosity around spiracles. Mesopleuron polished, area behind anterior margin setigerous-punctate; precoxal sulcus only indicated medially by a very shallow depression.

Wings. Forewing with areolet more or less 4-sided, vein r-m reduced to a transparent point and received onto vein r+3-SR, vein r much longer than vein 3-SR, arising from distal 1/2 of pterostigma, far behind the middle, placed at almost right angles to the pterostigma; meeting vein 2-SR at a 160 degree angle. Vein 1-R1 (metacarp) with distal end sharply defined, 1.9× as long as its distance from the apex of the marginal cell and 0.8× as long as pterostigma; pterostigma short and broad, 2.8× as long as wide; r:2-SR:length of pterostigma=8.0:7.0:28.0. Width of 1st discal cell: height of 1st discal cell = 20.5:17.5; 1-CU1:2-CU1:m-cu = 7.0:11.0:6.0. Hind wing with vannal lobe evenly covex, fringed with long setae throughout.

Legs. Long and thin. Hind coxa with dense fine punctures with interspaces smooth and shiny, with dense short fine setae medially and on posterior 1/3; dorsal surface also with dense fine punctures and dense short fine setae. Hind tibia swollen apically and only 0.7× as long as hind tarsus (38.0:49.7), outer surface without spines. Inner hind tibial spur longer than outer one, 0.6× as long as hind basitarsus (13.0:21.9); fourth tarsal segment much shorter than fifth tarsal segment (5.0:7.0); apical segment of the front tarsus without a spine. Tarsal claws simple.

Metasoma. Longer than mesosoma. T1 parallel-sided but roundly constricted distally, surface shiny with anterior 3/4 smooth and posterior 1/4 shallowly rugulose, with sparse fine setae dorsally and laterally; 1.1× as long as its width and 1.5× as long as T2; the percurrent median groove reduced on apex. T2 trapezoid, polished, with a slightly convex median field separated from other parts by two lateral sulci sharply narrowed on anterior half and parallel on posterior half, 0.5× as long as wide and 0.8× as long as T3. Tergites posterior to T2 more membranous, highly polished with a row of short fine setae before posterior margin, except the last two segments scattered with short fine setae. Ovipositor sheath slightly widened apically, decurved, with 2–3 blackened setae at apex. Hypopygium large, evenly sclerotised, polished, with very few long fine setae, apex not surpassing the last tergite.

Colour. Body black, metasoma brownish. Antennae yellowish brown at base and darkened toward apex to brown, pedicel brown. Mouthparts brownish yellow with palpi lighter. Tegula also brownish yellow. Legs almost virtually brownish yellow, except basal 4/5 of hind coxa, apex of hind femur and tibia and apex of claws brown. Tergites of abdomen evenly dark brown, basal sternites transparent and yellowish. Wings hyaline; veins brown, pterostigma, 1-R1(metacarp), submaginal vein and r vein darker.

Variation. Paratype with the same characteristics as holotype, but slightly different in colour pattern.

**Figures 9–16.**
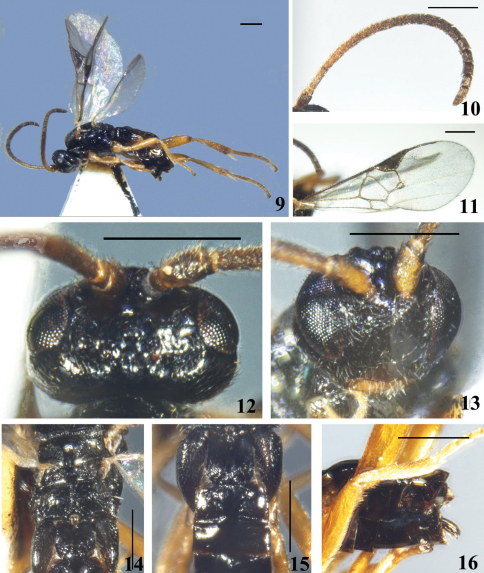
*Diolcogaster brevivena* Zeng & Chen, sp. n. **9** habitus, lateral view **10** antennae **11** fore wing **12** head, dorsal view **13** head, frontal view **14** scutellum and propodeum, dorsal view **15** T1–3, dorsal view **16** hypopygium and ovipositor sheaths, lateral view. Scale line = 0.5 mm.

#### Male.

 Unknown.

#### Host.

 Unknown.

#### Material examined.

 Holotype: ♀, Natural Park of Mt. Gaoligong (98°46'E, 24°49'N), Lujiangba, Baoshan, Yunnan, 2009. V. 10–11, legs. Wang Manman, No. 200903858. Paratype: 1♀, Natural Park of Mt. Gaoligong (98°46'E, 24°49'N), Lujiangba, Baoshan, Yunnan, 2009. V. 10–11, legs. Wang Manman, No. 200903862.

#### Etymology.

 The specific name “brevivena” derives from the Latin prefixion “brevi-” and noun “vena”, referring to the distinctly short metacarp.

#### Distribution.

 China (Yunnan).

#### Remarks.

 This species is similar to *Diolcogaster minuta* (Reinhard, 1880), but can be distinguished by T2 highly polished (the latter with T2 strongly rugose) and vein 1-R1 much longer than its distance from the apex of the radial cell (the latter with 1-R1 vein at most a little longer than its distance from the apex of the radial cell).

### Diolcogaster chaoi

(Luo & You, 2003)

http://species-id.net/wiki/Diolcogaster_chaoi

Figs 41–48

Caracallatus chaoi Luo and You 2003: 121. Holotype female, pinned with labels as follows: “1♀, Huishui, Guizhou, 1100M, 2001-X-24, legs. Luo Qinghuai” in Guizhou Normal University, Guiyang, China.
Diolcogaster chaoi : Luo and You 2005, 27: 50.

#### Material examined.

 1♀, Wanjia, Mayanghe (108°13'E, 28°35'N ~ 108°21'E, 28°41'N), Guizhou, 2007. IX. 27–30, legs. Zhu Lanlan, No. 200708137; 1♀, Huangguatuo, Mayanghe (108°13'E, 28°35'N ~ 108°21'E, 28°41'N), Guizhou, 2007. X. 1, legs. Liu Jingxian, No. 200709045; 1♀, Nanxi (103°50'E, 22°35'N ~ 103°57'E, 22°40'N), Hekou, Yunnan, 2003. VII. 20–21, legs. Xu Zaifu, No. 20055292; 1♀, Mt. Jianfengling (108°48'E, 18°41'N ~ 108°49'E, 18°42'N), Ledong, Hainan, 2007. VI. 5–7, light trap, legs. Liu Jingxian, No. 200703638; 1♂, Mt. Wuzhi (109°39'E, 18°51'N ~ 109°41'E, 18°54'N), Shuiman, Hainan, 2007. V. 15–20, legs. Weng Liqiong, No. 200804008; 1♀, Mt. Bawangling (109°02'E, 19°05'N ~ 109°04'E, 19°08'N), Changjiang, Hainan, 2007. VI. 9–10, legs. Liu Jingxian, No. 200703588; 1♀, Mt. Yinggeling (109°11'E, 18°49'N ~ 109°34'E, 19°08'N), Baisha, Hainan, 2008. XI. 18, legs. Tan Jiangli, No. 200805272; 1♂, Mt. Yinggeling (109°11'E, 18°49'N ~ 109°34'E, 19°08'N), Baisha, Hainan, 2008. XI. 18, legs. Tan Jiangli, No. 200805265; 1♀, Mt. Yinggeling (109°31'E, 19°04'N), Baisha, Hainan, 2007. V. 28-VI. 3, legs. Weng Liqiong, No. 200804192; 1♀, Mt. Yinggeling (109°11'E, 18°49'N ~ 109°34'E, 19°08'N), Baisha, Hainan, 2008. XI. 17, legs. Wan Manman, No. 200805447; 2♂♂, Mt. Yinggeling (109°11'E, 18°49'N ~ 109°34'E, 19°08'N), Baisha, Hainan, 2008. XI. 17, legs. Wan Manman, No. 200805453, 200805481; 1♂, Mt. Diaoluo (109°53'E, 18°39'N ~ 109°58'E, 18°42'N), Lingshui, Hainan, 2006. VII. 16–17, legs. Liu Jingxian, No. 200802233; 1♂, Yacheng (109°09'E, 18°09'N ~ 109°31'E, 18°26'N), Sanya, Hainan, 2008. XI. 21, legs. Wang Manman, No. 200805044; 1♀, Mt. Qingyun (118°54'E, 25°43'N ~ 119°01'E, 25°48'N), Yongtai, Fujian, 2002. IX. 18, legs. Yu Xiaoxia, No. 20023506.

**Host**. Unknown.

#### Distribution.

 China (Fujian, Hainan, Guizhou, Yunnan).

### Diolcogaster grammata

Zeng & Chen sp. n.

urn:lsid:zoobank.org:act:5F33B360-090C-4A81-9872-B094EB0DB884

http://species-id.net/wiki/Diolcogaster_grammata

Figs 17–24

#### Description.

 Female. Body length 3.0 mm, fore wing length 3.1 mm.

Head. Rather large. Oval in anterior view, with antennal sockets high above middle level of eyes; transverse in dorsal view, 1.5× as wide as long and shorter than that of mesoscutum, shiny with dense short setae all over. Ocelli small in a low, very wide triangle, the transverse, posterior tangent to the anterior ocellus just cutting the posterior pair. POL:OD:OOL=5.0:3.8:7.7. Vertex strongly convex, and sharply constricted to occiput, densely rugose-punctate; frons densely rugose-punctate except the polished area behind scape and pedicel; face coarsely rugose-punctate, without median keel; its width 1.4× as high as eye and clypeus combined (25.6:18.0); eyes of moderate size, inner margins of eyes parallel, 1.8× as high as wide (23.8:13.1); temple and gena striate-punctate. Mouth opening wide; clypeus densely rugose; tentorial pits small, distance between tentorial pits 3.4× as long as distance from pit to eye margin (10.8:3.2); malar space short, 0.2× as long as eye height. Antennae longand with the preapical segment fully 2.5× as long as wide; scape short; flagellomeres thinner, tappered distally, with bristly setosity, with placodes arranged regularly in 2 ranks; apical segment slightly longer than preapical one. Flagellomere proportion: 2 L/W=3.1, 8 L/W=3.2, 14 L/W=2.0; L 2/14=1.7; W 2/14=1.1.

Mesosoma. Pronotum shiny, with a distinct foveate ventral furrow laterally. Mesoscutum shiny, with uneven, dense puntures or rugose-punctures, more in evidence anteriorly and along imaginary course of the notauli, with dense short setae all over. Disc of scutellum shiny but densely rugose-punctate, also with dense short setae all over; its rugose tip at middle widely interrupting the posterior, polished band of scutellum, separated from disc by a short transverse keel, with dense short setae. Lateral part of the polished band distinctly broadened anteriorly. Anterior margin of the postscutellum closely applied to the posterior margin of the scutellum so that, laterally, the phragma of scutellum is completely hidden. Scutellar sulcus shallow, with a few short longitudinal carinae, its width 1.1× as long as scutellum (18.0:15.9). Propodeum shiny, coarsely reticulate-rugose except anterior area behind the anterior margin, with a weak median keel. Mesopleuron polished medially, with dense punctures but interspaces shiny; precoxal sulcus broad, indistinct, shallow with few punctures.

Wings. Forewing with areolet virtually 3-sided; r (1st abscissa of radius) arising from distal 1/2 of pterostigma, far behind the middle, placed more nearly at right angles on the pterostigma, meeting vein 2-SR at a 145 degree angle. Vein 1-R1 (metacarp) with distal end sharply defined and almost reaching to apex of marginal cell, 7.0× as long as its distance from the apex of the marginal cell and as long as pterostigma, pterostigma 2.8× as long as wide; r:2-SR: length of pterostigma=12.0:12.0:34.0. Width of 1st discal cell:height of 1st discal cell =27.0:22.0; 1-CU1:2-CU1:m-cu=10.5:9.5:11.5. Hind wing narrow, with vannal lobe beyond its widest part convex, fringed with long setae throughout.

Legs. Long and thin. Hind coxa large, shiny with densely punctate surface anteriorly and coarsely reticulate-punctate surface posteriorly, reaching to posterior margin of T3. Hind tibia gradually swollen toward apex and about 0.8× as long as hind tarsus (53.0:69.5). Inner hind tibial spur much longer than outer one and 0.8× as long as hind basitarsus (23.0:30.0); fourth tarsal segment shorter than fifth tarsal segment (9.0:12.0); apical segment of the front tarsus without a spine. Tarsal claws long, simple.

Metasoma. Longer than mesosoma. T1 very long and narrow, linear, parallel-sided, with complete longitudinal groove that is reduced apically, smooth on anterior 1/3 and setigerous punctate on posterior 2/3, 3.1× as long as its width and 2.5× as long as T2. T2 strongly transverse, with a distinctly raised pentagonal median field that is sharply-sided and with smooth and shiny surface, 0.5× as long as its greatest width and 0.7× as long as T3; area beside median field setose, smooth and shiny. Tergites posterior to T2 smooth and shiny, more membranous; only last 3 tergites with a few short fine setae. Ovipositor sheath thin, decurved, with 2–3 blackened setae at apex, long, spatulate. Hypopygium small, evenly sclerotised, smooth and densely setose posteriorly, not surpassing the last tergite.

Colour. Body bright yellow to black; head and thorax black, metasoma almost bright yellow, except the median field of T2 and ovipositor sheaths brown, last 4 tergites light brown medially. Flagellum yellowish brown basally, thickened toward apex, scape and pedicel bright yellow, brownish laterally. Mouthparts yellow, ventral margin of clypeus and mandible brownish; palpi whitish. Tegula whitish yellow. Fore and mid legs bright yellow, whitish basally. Hind coxa and femur black, except the extreme apex of coxa and basal area of femur yellow, trochanter also yellow; hind tibia and tarsus brown except that basal half of tibia and tibial spurs are bright yellow and apical tarsus is yellowish. Wings hyaline; veins brown but greyish on basal half and on metacarp, pterostigma brown, with basal spot grey.

Variation. Paratype with the same characteristics as holotype, but more or less transparent.

**Figures 17–24.**
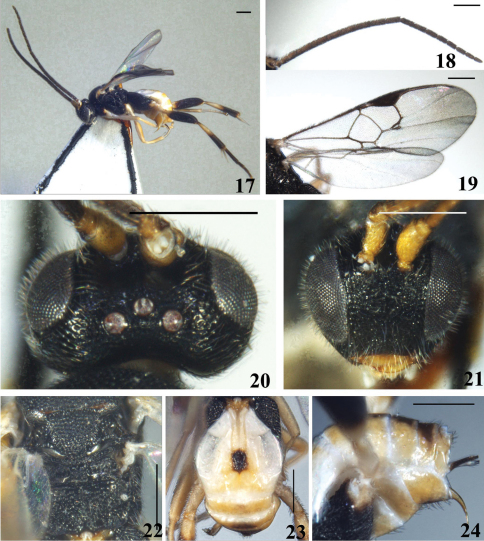
*Diolcogaster grammata* Zeng & Chen, sp. n. **17** habitus, lateral view **18** antennae **19** fore wing **20** head, dorsal view **21** head, frontal view **22** scutellum and propodeum, dorsal view **23** T1–3, dorsal view **24** hypopygium and ovipositor sheaths, lateral view. Scale line = 0.5 mm.

#### Male.

 Unknown.

#### Host.

 Unknown.

#### Material examined.

 Holotype: ♀, Mt. Wuzhi (109°39'E, 18°51'N ~ 109°41'E, 18°54'N), Shuiman, Hainan, 2007. V. 16–20, Liu Jingxian, No. 200703126. Paratype: 1♀, Mt. Nankun (113°51'E, 23°37'N ~ 113°53'E, 23°38'N), Longmen, Guangdong, 2003. VII. 14–15, Xu Zaifu, No. 20050313.

#### Etymology.

 The specific name “*grammata*” derives from the Latin adjective “grammatus”, referring to the narrow, linear T1.

#### Distribution.

 China (Hunan, Guangdong, Hainan).

#### Remarks.

 This species is similar to *Diolcogaster xanthaspis* (Ashmead, 1900), but can be distinguished by the well-defined median field on T2 (the latter with poorly defined median field on T2+T3); 1-CU1 slightly longer than 2-CU1 (the latter with 1-CU1 as long as 2-CU1); and the ovipositor sheath with modified apical setae (the latter without).

### Diolcogaster ineminens

Zeng & Chen sp. n.

urn:lsid:zoobank.org:act:65742BCC-8FF2-44C8-8011-660622E13D61

http://species-id.net/wiki/Diolcogaster_ineminens

Figs 25–32

#### Description.

 Female. Body length 3.8 mm, fore wing length 4.5 mm.

Head. Oval in anterior view, with antennal sockets high above middle level of eyes; transverse in dorsal view, 1.5× as wide as long and almost as long as mesoscutum, with very dense short setae except the sharply constricted and highly polished area behind lateral ocelli. Ocelli small, in a low triangle, the transverse, posterior tangent to the anterior ocellus just cutting the posterior pair. POL:OD:OOL=6.8:5.0:6.0. Vertex shiny, convex, with distantly discrete large punctures out of ocular area; frons polished and without setae in large part but densely rugose-punctate and setose adjacent to eye margin; face slightly convex, also shiny, densely setigerous-punctate, with an indistinct longitudinal median keel that neither extend to dorsal margin of face nor extend to clypeus, width of face 0.7× as high as eye and clypeus combined (22.7:33.3); eyes large, inner margin of eyes parallel, 1.6× as high as wide (30.0:18.5); temple and gena shiny, densely but finely transversely striate-punctate, with appressed setae. Clypeus densely rugulose, slightly convex; tentorial pits large, distance between tentorial pits 2.5× as long as distance from pit to eye margin; malar space short, 0.2× as long as eye height. Antennae long and thin with the preapical segment more than 1.75× as long as wide; flagellomeres not thickened apically, without bristly setosity, with placodes arranged regularly in 2 ranks; apical segment broken. Flagellomere proportion: 2 L/W=4.6 , 8 L/W=2.6, 14 L/W=1.8; L 2/14=2.1; W 2/14=0.5.

Mesosoma. Pronotum flattened, shiny, with a broad and deep foveate ventral furrow laterally, densely setigerous-punctateabove. Mesoscutum shiny, with very dense and evenly departed punctures, more or less rugose-punctate posteromedially, with dense short setae all over; notauli not impressed. Disc of scutellum also shiny, covex, more densely punctate than mesoscutum, setose; its rugose tip at middle widely interrupting the posterior, polished band of scutellum. Lateral part of the polished band distinctly convex anteriorly. Anterior margin of the postscutellum closely applied to the posterior margin of the scutellum so that, laterally, the phragma of scutellum is completely hidden. Scutellar sulcus deep and broad, with few strong longitudinal carinae, its width almost as long as scutellum (24.0:25.0). Propodeum strongly and coarsely rugose, with strong costulae around spiracles, with a strong complete median keel. Mesopleuron polished above precoxal sulcus, with very shallow and distantly discrete punctures on ventral half, with setigerous-punctures anteriorly and below precoxal sulcus; precoxal sulcus very shallow, indistinct, densely and shallowly punctate.

Wings. Forewing with a large 3-sided areolet; vein r arising from distal 1/2 of pterostigma, far behind the middle, very obliquely placed on the pterostigma, meeting vein 2-SR at 110 degree angle. Vein 1-R1 (metacarp) with distal end sharply defined, 5.8× as long as its distance from the apex of the marginal cell and 1.4× as long as pterostigma, pterostigma 3.4× as long as wide; r:2-SR:length of pterostigma=15.0:11.0:37.0. Width of 1st discal cell:height of 1st discal cell =37.0:26.0; 1-CU1:2-CU1:m-cu=10.0:20.0:13.0. Hind wing with vannal lobe evenly covex, fringed with long setae throughout.

Legs. Long and strong. Hind coxa large, shiny, with outer and dorsal surface very densely rugulose-punctate, evenly setose all over; just reaching to anterior margin of T3. Hind tibia swollen medially and then slightly narrowed apically, 0.9× as long as hind tarsus (85.0:93.5), outer surface without spines. Inner hind tibial spurs longer than outer one, 0.5× as long as hind basitarsus (22.0:42.5); fourth tarsal segment shorter than fifth tarsal segment (9.0:14.0); apical segment of the front tarsus without a spine. Tarsal claws simple.

Metasoma. Shorter than mesosoma. T1, T2 and anterior half of T3 strongly sclerotised. T1 parallel-sided, roundly constricted at apex, longitudinally strigous-punctate all over; scattered with appressed setae, denser on posterior 1/4; bearing a distinct median groove with smooth edges; 1.9× as long as its width and 1.2 as long as T2. T2 longitudinally aciculate with interspaces rugose and without setae, without trace of median field, with anterior margin back off each side out of the middle, 0.8× as long as wide and twice as long as T3. T3 subrectangular, with posterior half less sclerotised, also longitudinally aciculate but more finely than T2. Tergites posterior to T3 membranous, polished, sparsely setose. Ovipositor sheath with even width, slightly decurved, without modified apical setae. Hypopygium large, evenly sclerotised, polished with sparse long fine setae apically, apex not surpassing the last tergite.

Colour. Body black, metasoma mostly yellowish brown, more or less transparent. Antennae with scape, pedicel, apical 1/3 of flagellum and outer surface of basal 1/3 of flagellum brown, middle 1/3 and outer surface of basal 1/3 of flagellum white. Labrum dark brown and lightened downwards, labium bright yellow with brown margin, palpi brown. Tegula yellowish brown. Fore and middle legs yellow at base, darkened apically to brown. Hind coxa black, except the reddish apex; trochanter yellow; femur and tibia dark brown except the reddish basal halves; tarsi brown, gradually and slightly lightened apically; tibial spurs whitish yellow. T1 and T2 dark brown, T3 also dark brown anteriorly, then gradually lightened apically to yellowish brown and more or less transparent; apical segment and ovipositor sheaths also brown. Wings hyaline; veins and pterostigma brown, except the basal pale spot of pterostigma.

Variation. Individuals from Fujian with basal 1/3 of flagellum brown just like inner surface.

**Figures 25–32.**
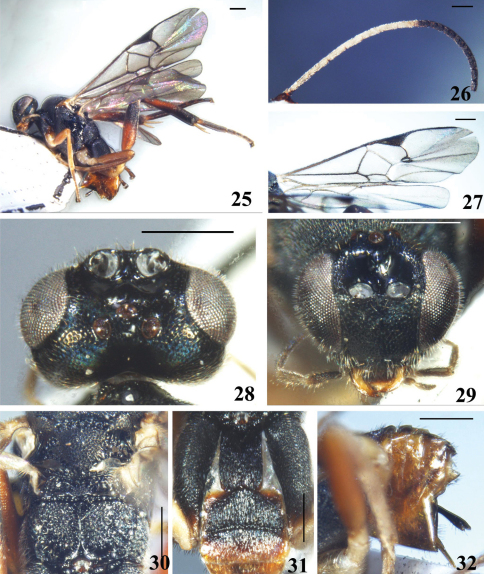
*Diolcogaster ineminens* Zeng & Chen, sp. n. **25** habitus, lateral view **26** antennae **27** fore wing **28** head, dorsal view **29** head, frontal view **30** scutellum and propodeum, dorsal view **31** T1–3, dorsal view **32** hypopygium and ovipositor sheaths, lateral view. Scale line = 0.5 mm.

#### Male.

 Unknown.

#### Host.

 Unknown.

#### Material examined.

 Holotype: ♀, Mt. Nanling (112°59'E, 24°53'N ~ 113°05'E, 24°56'N), Ruyuan, Guangdong, 2004. VIII. 4, legs. Xu Zaifu, No. 20049886. Paratype: 2♀♀, Mt. Longqi (117°37'E, 26°15'N ~ 117°39'E, 26°16'N), Jiangle, Fujian, 1991. VII. 8, legs. Liu Changming, No. 20006911, 20006940; 1♀, Mt. Gutian (118°07'E, 29°14'N ~ 118°10'E, 29°16'N), Kaihua, Zhejiang, 2005. VII. 3, legs. Chen Xuexin, No. 200616278; 1♀, Mt. Gutian (118°07'E, 29°14'N ~ 118°10'E, 29°16'N), Kaihua, Zhejiang, 2005. VII. 2, legs. Wu Qiong, No. 200616765; 1♀, Mt. Gutian (118°07'E, 29°14'N ~ 118°10'E, 29°16'N), Kaihua, Zhejiang, 2005. VII. 3, legs. Wu Qiong, No. 200616991.

#### Etymology.

 The specific name “ineminens” derives from the Latin prefixion “in-” and adjective “eninens”, referring to T2 without a raised median field.

#### Distribution.

 China (Zhejiang, Fujian, Guangdong).

#### Remarks.

 This species is similar to *Diolcogaster abdominalis* (Nees, 1834), but can be distinguished by the ovipositor sheath without modified seta (the latter has ovipositor sheath with a row of four fine black setae, arising from the lower margin); pterostigma emitting radius much distal to middle (the latter with pterostigma emitting radius at most slightly beyond middle); and radial cell of fore wing normal (the latter with radial cell abruptly narrowed apically).

### Diolcogaster laetimedia

Zeng & Chen sp. n.

urn:lsid:zoobank.org:act:09B64969-36A6-405B-9B9D-0ACEDC6C6F05

http://species-id.net/wiki/Diolcogaster_laetimedia

Figs 33–40

#### Description.

 Female. Body length 2.9 mm, fore wing length 3.3 mm.

Head. Rather large, subtriangular in anterior view, with antennal sockets high above middle level of eyes; oval in dorsal view, 1.5× as wide as long and a little longer than mesoscutum, strongly concave behind ocular area, scattered with very short setae except the sharply constricted and highly polished area behind lateral ocelli. Ocelli small, in a low triangle, but the transverse, posterior tangent to the anterior ocellus a little before the posterior pair. POL:OD:OOL=5.3:3.4:4.7. Vertex shiny, convex, ocular area polished, with distantly discrete fine punctures out of ocular area; frons polished and without setae in large part but densely rugulose and setose adjacent to eye margin; face slightly convex, also shiny, densely but shallowly punctate, longitudinal median keel only indicated on dorsal 1/4, width of face 0.7× as high as eye and clypeus combined (19.0:26.8); eyes rather large, inner margin of eyes parallel, 1.6× as high as wide (28.0:17.8); temple and gena shiny, densely but feebly transversely striate-punctate, with dense appressed setae. Clypeus densely rugulose, slightly convex; tentorial pits of moderate size, distance between tentorial pits 2.4× as long as distance from pit to eye margin; malar space very short, only 0.2× as long as eye height. Antennae long and thin, with the preapical segment fully 2.5× longer than wide; flagellomeres not thickened apically, without bristly setosity, with placodes arranged regularly in 2 ranks; apical segment distinctly longer than preapical one. Flagellomere proportion: 2 L/W=5.0, 8 L/W=2.4, 14 L/W=1.8; L 2/14=2.1; W 2/14=0.4.

Mesosoma. Pronotum shiny, with a shallowly striate ventral furrow laterally, densely and discretely punctate above. Mesoscutum shiny, with very dense and evenly discrete punctures, with dense short setae all over; notauli not impressed. Disc of scutellum also shiny, covex, as densely punctate as mesoscutum; its rugose tip at middle widely interrupting the posterior, polished band of scutellum. Lateral part of the polished band distinctly convex anteriorly. Anterior margin of the postscutellum closely applied to the posterior margin of the scutellum so that, laterally, the phragma of scutellum is completely hidden. Scutellar sulcus deep but narrow, with few strong longitudinal carinae, its width as long as scutellum (16.0:16.0). Propodeum with surface coarsely rugose on anterior 2/3 and polished on posterior 1/3, with strong costulae around spiracles and dull inside, with a very strong complete median keel. Mesopleuron polished above precoxal sulcus but with dense setigerous-punctures anteriorly and below precoxal sulcus; precoxal sulcus indicated by a shallow depression, with few punctures.

Wings. Forewing with the areolet is 4-sided, vein r(1st abscissa of the radius) much longer than vein 3-SR (2nd abscissa of the radius), arising from distal 1/2 of pterostigma, far behind the middle, very obliquely placed on the pterostigma, meeting vein 2-SR at 110 degree angle. Vein 1-R1 (metacarp) with distal end sharply defined, 4.8× as long as its distance from the apex of the marginal cell and 1.2× as long as pterostigma; pterostigma 3.0× as long as wide; r:2-SR:length of pterostigma=10.5:7.0:31.0. Width of 1st discal cell:height of 1st discal cell =27.0:19.0; 1-CU1:2-CU1:m-cu=8.2:16.0:10.5. Hind wing with vannal lobe evenly convex, fringed with long setae throughout.

Legs. Long and strong. Hind coxa large, reaching to posterior margin of T3, shiny, with dense and evenly discrete punctures, evenly setose all over. Hind tibia swollen apically, 0.8× as long as hind tarsus (58.0:76.0), outer surface with very sparse fine spines. Inner hind tibial spur longer than outer one, 0.5× as long as hind basitarsus (18.0:34.5); fourth tarsal segment shorter than fifth tarsal segment (6.9:10.0); apical segment of the front tarsus without a spine. Tarsal claws simple.

Metasoma. Shorter than mesosoma. T1 slightly narrowed toward apex, shiny, finely rugulose and with distinct median groove on anterior 3/5, densely rugose and with appressed setae on posterior 2/5, 2.0× as long as its width and twice as long as T2. T2 transverse, narrowed apically, anterior corner distinctly projecting anteriorly, longitudinally aciculate with interspaces rugose and without setae, without trace of median field, with very sparse short fine setae, 0.7× as long as wide and as long as T3. T3 subrectangular, very finely longitudinally aciculate, also with very few fine setae. Tergites posterior to T3 membranous, polished, sparsely setose. Ovipositor sheath slightly widened apically, straight, without modified setae at apex. Hypopygium large, evenly sclerotised, polished with sparse long fine setae, apex not surpassing the last tergite.

Colour. Body black, somewhat brownish; metasoma dark brown, lighter and more or less transparent ventrally. Antennae brown except for 3–4 white middle flagellomeres with scape and pedicel transparent ventrally. Mouthparts brownish yellow, ventral margin and apex of labium brown, palpi whitish yellow. Tegula brown. Legs almost brown, with fore and middle legs lighter, except the trochanters, fore and middle coxae and ventral part of hind coxa pale yellow; hind femur yellow at base; tibial spurs whitish yellow. Wings hyaline; veins and pterostigma brown, except the basal pale spot of pterostigma.

Variation. Some individuals from Hainan Island with legs and metasoma lighter coloured, indicated by yellow colour.

**Figures 33–40.**
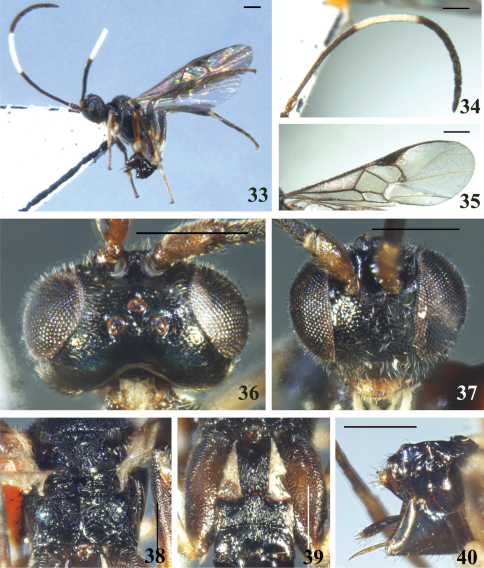
*Diolcogaster laetimedia* Zeng & Chen, sp. n. **33** habitus, lateral view **34** antennae **35** fore wing **36** head, dorsal view **37** head, frontal view **38** scutellum and propodeum, dorsal view **39** T1–3, dorsal view **40** hypopygium and ovipositor sheaths, lateral view. Scale line = 0.5 mm.

**Figures 41–48.**
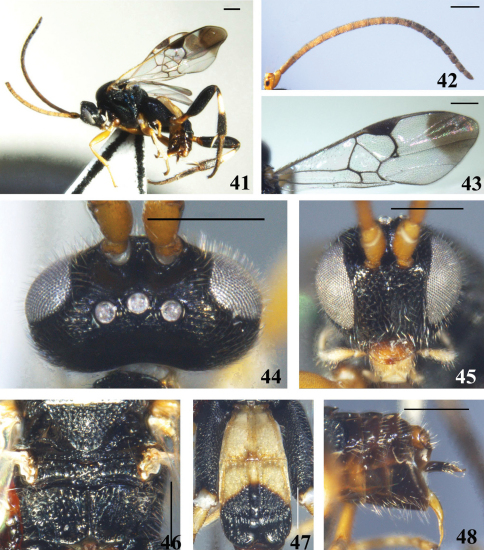
*Diolcogaster chaoi* (Luo & You, 2003) **41** habitus, lateral view **42** antennae **43** fore wing **44** head, dorsal view **45** head, frontal view **46** scutellum and propodeum, dorsal view **47** T1–3, dorsal view **48** hypopygium and ovipositor sheaths, lateral view. Scale line = 0.5 mm.

**Figures 49–56.**
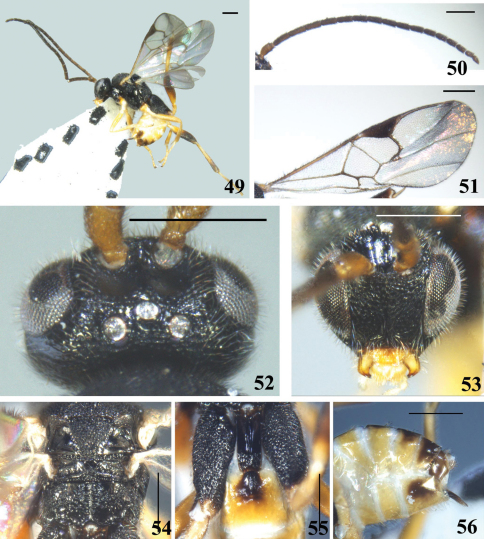
*Diolcogaster pluriminitida* Zeng & Chen, sp. n. **49** habitus, lateral view **50** antennae **51** fore wing **52** head, dorsal view **53** head, frontal view **54** scutellum and propodeum, dorsal view **55** T1–3, dorsal view **56** hypopygium and ovipositor sheaths, lateral view. Scale line = 0.5 mm.

#### Male.

 Unknown.

#### Host.

 Unknown.

#### Material examined.

 Holotype: ♀, Mt. Nanling (112°59'E, 24°53'N ~ 113°05'E, 24°56'N), Ruyuan, Guangdong, 2004. V. 8, legs. Xu Zaifu, No. 20049497. Paratype: 1♀, Mt. Nanling (112°59'E, 24°53'N ~ 113°05'E, 24°56'N), Ruyuan, Guangdong, 2004. V. 8, legs. Xu Zaifu, No. 20049461; 2♀♀, Mt. Chebaling (114°14'E, 24°43'N ~ 114°16'E, 24°44'N), Shixing,Guangdong, 2002. IV. 19, legs. Xu Zaifu, No. 20050439, 20050825; 2♀♀, Mt. Chebaling (114°14'E, 24°43'N ~ 114°16'E, 24°44'N), Shixing, Guangdong, 2003. VIII. 21, legs. Xu Zaifu, No. 20051996, 20052381; 1♀, Mt. Gutian (118°07'E, 29°14'N ~ 118°10'E, 29°16'N), Kaihua, Zhejiang, 2005. VII. 2, legs. Wu Qiong, No. 200616858; 1♀, Mt. Gutian (118°07'E, 29°14'N ~ 118°10'E, 29°16'N), Kaihua, Zhejiang, 2005. VII. 3, legs. Wu Qiong, No. 200616985; 1♀, Mt. Gutian (118°07'E, 29°14'N ~ 118°10'E, 29°16'N), Kaihua, Zhejiang, 2005. VII. 3, legs. Chen Xuexin, No. 200616274; 1♀, Mt. Diaoluo (109°53'E, 18°39'N ~ 109°58'E, 18°42'N), Lingshui, Hainan, 2007. V. 28-VI. 1, legs. Zeng Jie, No. 200806652; 1♀, Tianchi, Mt. Jianfengling (108°48'E, 18°41'N ~ 108°49'E, 18°42'N), Ledong, Hainan, 2006. VII. 12–15, legs. Weng Liqiong, No. 200803277; 1♀, Tianchi, Mt. Jianfengling (108°48'E, 18°41'N ~ 108°49'E, 18°42'N), Ledong, Hainan, 2008. XI. 24, legs. Tan Jiangli, No. 200806060; 1♀, Letu (117°13'E, 24°53'N ~ 117°14'E, 24°54'N), Nanjing. Fujian, 1991. V. 23, legs. Liu Changming, No. 20006002.

#### Etymology.

 The specific name “laetimedia” derives from the Latin adjective “laetus” and noun “media”, referring to middle of antennae with bright white color.

#### Distribution.

 China (Zhejiang, Fujian, Guangdong, Hainan).

#### Remarks.

 This species is similar to *Diolcogaster connexus* (Nees, 1834), but can be distinguished by the pterostigma emitting radius much distal to middle (the latter with pterostigma emitting radius at most slightly beyond middle); T2 as long as T3 (the latter with T2 much shorter than T3); and tergites posterior to T3 only sparsely setose (the latter with metasoma with dense setae, especially on T3).

### Diolcogaster perniciosa

(Wilkinson, 1929)

http://species-id.net/wiki/Diolcogaster_perniciosa

Figs 81–88

Microgaster perniciosa Wilkinson 1929, 77: 122. Holotype female, pinned with labels as follows: “Australia: Victoria” undated (London, ♀, 3.c. 1310).
Protomicroplitis perniciosa : Nixon 1965: 248.

#### Material examined.

 1♀, Mt. Longqi (117°37'E, 26°15'N ~ 117°39'E, 26°16'N), Jiangle, Fujian, 1991. VII. 1, legs. Liu Changming, No. 20006438; 1♀, West Mt. Tianmu (119°23'E, 30°20'N ~ 119°24'E, 30°20'N), Linan, Zhejiang, 1995. V. 9, legs. Du Yuzhou, No. 977839; 1♀, Mangkuan (98°51'E, 25°26'N ~ 98°53'E, 25°27'N), Baoshan, Yunnan, 2006. VII. 18, legs. Zeng Jie, No. 200801846; 1♀, Shaba (98°42'E, 25°23'N ~ 98°43'E, 25°24'N), Jietou, Tengchong, Yunnan, 2009. V. 13, legs. Wang Manman, No. 200904443; 1♀, Langsu, Tongbiguan (97°38'E, 24°37'N), Yingjiang, Yunnan, 2009. V. 16, legs. Wang Manman, No. 200905077; 1♀, Langsu, Tongbiguan (97°38'E, 24°37'N), Yingjiang, Yunnan, 2009. V. 16, legs. Zeng Jie, No. 200904674; 1♀, Mulianhuatang (97°39'E, 24°36'N), Tongbiguan, Yingjiang, Yunnan, 2009. V. 20, legs. Zeng Jie, No. 200905319; 1♀, Mt. Cangshan (100°10'E, 25°38'N), Dali, Yunnan, 2009. V. 25, legs. Wang Manman, No. 200904181; 1♀, Xiangshuwan (107°13'E, 28°12'N), Kuankuoshui Natural Reserve, Suiyang, Guizhou, 2010. VI. 3, legs. Chai Hongfei, No. 201004207; 3♀♀, Xiangshuwan (107°13'E, 28°12'N), Kuankuoshui Natural Reserve, Suiyang, Guizhou, 2010. VI. 4, legs. Tan Jiangli, No. 201002737, 201002748, 201002749; 2♀♀, Kuankuoshui Natural Reserve (107°14'E, 28°22'N ~ 107°15'E, 28°22'N), Suiyang, Guizhou, 2010. VI. 5, legs. Tang Pu, No. 201001371, 201001662; 1♀, the core area of Kuankuoshui Natural Reserve (107°15'E, 28°22'N), Suiyang, Guizhou, 2010. VI. 6–7, legs. Tan Jiangli, No. 201000857; 1♀, Shilingou (107°15'E, 28°22'N), Kuankuoshui Natural Reserve, Suiyang, Guizhou, 2010. VI. 9, legs. Tan Jiangli, No. 201005532; 1♀, Jiulongshan (106°35'E, 26°08'N), Huishui, Guizhou, 2010. VI. 11, legs. Zeng Jie, No. 201003863.

#### Host.

*Nyctemera amica*, *Nyctemera annulata*, *Nyctemera* [*Senecio jacobaea*]; *Spilosoma glatignyi* (Arctiidae) (Yu & van Achterberg, 2004).

#### Distribution.

 China (Zhejiang, Fujian, Guizhou, Yunnan); Austrialia, New Zealand.

#### Remarks.

This species is record from China for the first time.

### Diolcogaster pluriminitida

Zeng & Chen sp. n.

urn:lsid:zoobank.org:act:C0DCEB36-9910-42F3-9668-A4A48A807250

http://species-id.net/wiki/Diolcogaster_pluriminitida

Figs 49–56

#### Description.

 Female. Body length 3.0 mm, fore wing length 2.9 mm.

Head. Oval in anterior view, with antennal sockets high above middle level of eyes; transverse in dorsal view, 1.5× as wide as long and almost as long as mesoscutum, with very dense short fine setae except for the sharply constricted and highly polished area behind lateral ocelli. Ocelli small, in a low and wide triangle, the transverse, posterior tangent to the anterior ocellus deeply cutting the posterior pair. POL:OD:OOL=6.2:4.0:6.7. Vertex vey shiny, strongly convex, finely transversely striate; frons polished and without setae in large part but densely rugulose and setose adjacent to eye margin; face slightly convex, also very shiny, densely strigose-punctate, transversely striate just below antennal sockets, without any trace of median keel, width of face 0.8× as high as eye and clypeus combined (17.8:23.7); eyes of moderate size, inner margin of eyes parallel, 1.8× as high as wide (22.3:12.7); temple and gena very shiny, sparsely transversely striate, with appressed long setae. Clypeus slightly convex, densely rugulose, with ventral margin slightly excavated medially; tentorial pits small, distance between tentorial pits 2.5× as long as distance from pit to eye margin; malar space very short, 0.2× as long as eye height. Antennae long and thin, with the preapical segment about 1.3× as long as wide; flagellomeres not thickened apically, with bristly setosity, with placodes arranged regularly in 2 ranks; apical segment also as long as preapical one. Flagellomere proportion: 2 L/W=3.2, 8 L/W=3.7, 14 L/W=2.2; L 2/14=1.6; W 2/14=0.6.

Mesosoma. Pronotum shiny, with a broad and shallowly foveate ventral furrows laterally. Mesoscutum shiny, with dense but discrete punctures that denser and so that duller in the middle, with dense short fine setae all over; notauli not impressed. Disc of scutellum also shiny, as densely and discretely punctate as mesoscutum; its rugose tip at middle widely interrupting the posterior, polished band of scutellum. Lateral part of the polished band distinctly convex anteriorly. Anterior margin of the postscutellum closely applied to the posterior margin of the scutellum so that, laterally, the phragma of scutellum is completely hidden. Scutellar sulcus shallow, with few longitudinal carinae, its width 1.3× as long as scutellum (21.0:16.3). Propodeum very shiny, with a strong complete median keel, smooth with very few discrete large punctures, with very short transverse carina each side along the median keel and dense rugulosity around spiracles. Mesopleuron polished in large part, setigerous-punctate anteriorly and below precoxal sulcus; precoxal sulcus shallow, indistinct, setigerous-punctate.

Wings. Forewing with the very small slit-like areolet, 3-sided; vein r-m united with vein 2-SR at a considerable distance from the junction of vein 2-SR and the vein r+3-SR; vein r arising from distal 1/2 of pterostigma, far behind the middle, very obliquely placed on the pterostigma; meeting vein 2-SR at a right angle. Vein 1-R1 (metacarp) with distal end sharply defined, 4.4× as long as its distance from the apex of the marginal cell and as long as pterostigma; pterostigma broad, 2.7× as long as wide; r:2-SR:length of pterostigma=11.5:10.0:32.0. Width of 1st discal cell:height of 1st discal cell =25.0:22.0; 1-CU1:2-CU1:m-cu=11.0:10.0:9.5. Hind wing with vannal lobe evenly convex, fringed with long setae throughout.

Legs. Long and thin. Hind coxa with outer surface densely and discretely punctate with interspaces very shiny, dorsal surface densely rugulose and striate-rugulose, reaching to the middle of T3, setose all over. Hind tibia swollen apically and only 0.8× as long as hind tarsus (48.5:63.8), outer surface without spines. Inner hind tibial spur longer than outer one, 0.7× as long as hind basitarsus (20.0:28.0); fourth tarsal segment almost as long as fifth tarsal segment (7.8:8.0); apical segment of the front tarsus without a spine. Tarsal claws simple.

Metasoma. Longer than mesosoma. T1 strongly sclerotised, narrow, parallel-sided but slightly narrowed distally on posterior half, with few long setae laterally; surface with anterior 3/4 polished and posterior 1/4 rugulose with fine setae, the distinct median groove reduced on posterior 1/4; 2.4× as long as its width and 2.4× as long as T2. T2 polished and without setae, with a slightly swollen median field, ill-defined, 0.4× as long as wide and 0.7× as long as T3. Tergites posterior to T2 membranous, highly polished with a row of short fine setae before posterior margin, except the last segment scattered with short fine setae. Ovipositor sheath with even width, slightly decurved, with 2 blackened setae at apex. Hypopygium of moderate size, evenly sclerotised, polished, with dense short setae all over, apex not surpassing the last tergite.

Colour. Body black, metasoma mostly bright yellow. Antennae evenly brown except scape and pedicel yellow. Mouthparts yellow with palpi whitish. Tegula bright yellow. Fore and middle legs virtually bright yellow. Hind coxa wholly black; trochanter yellow, base of femur and basal 3/4 of tibia also yellow but gradually darkened to brown apically; tarsi and tibial spurs whitish brown. T1 dark brown, the indistinct median field of T2 also brown but lightened outwards to bright yellow, apical 3 segments and ovipositor sheaths also brown, T5, T6 and lateral part of hypopygium brownish. Wings hyaline; veins and pterostigma light brown, somewhat pale.

Variation. Some individuals from Zhejiang and Guizhou have ovipositor sheath only finely setose, without apical modified seta; metasoma mostly yellow, but size of brownish part of dorsal surface and hypopygium variable; the ill-defined median field of T2 hardly impressed in some specimens.

#### Male.

 Unknown.

#### Host.

 Unknown.

#### Material examined.

 Holotype: ♀, Wanjia, Mayanghe (108°13'E, 28°35'N ~ 108°21'E, 28°41'N), Guizhou, 2007. IX. 27–30, legs. Liu Jingxian, No. 200708869. Paratype: 1♀, Mt. Huping (110°45'E, 30°02'N ~ 110°55'E, 30°07'N), Shimen, Hunan, 2009. VII. 12, legs. Ma Li, No. 200901491; 1♀, Mt. Huping (110°45'E, 30°02'N ~ 110°55'E, 30°07'N), Shimen, Hunan, 2009. VII. 12, legs. Zeng Jie, No. 200900730; 1♀, Mt. Tongledashan (111°20'E, 23°07'N ~ 111°29'E, 23°14'N), Yunan, Guangdong, 2003. VIII. 12–13, legs. Xu Zaifu, No. 20054555; 1♀, Mt. Leigong (118°03'E, 26°21'N ~ 118°15'E, 26°25'N), Fangxiang, Guizhou, 2005. VI. 2–3, legs. Liu Jingxian, No. 200605872; 1♀, West Mt. Tianmu (119°23'E, 30°20'N ~ 119°24'E, 30°20'N), Linan, Zhejiang (Malaise trap), 1998. IX. 26, legs. Zhao Mingshui, No. 20002719.

#### Etymology.

 The specific name “pluriminitida” derives from the Latin adjective “plurimus” and adjective “nitidus”, referring to large part of T1 polished.

#### Distribution.

 China (Zhejiang, Hunan, Guangdong, Guizhou).

#### Remarks.

 This species is similar to *Diolcogaster xanthaspis* (Ashmead, 1900), but can be distinguished by the T2 with only ill-defined slightly swollen median field that with even width (the latter T2+3 with distinct but rather poorly defined median field that is slightly wider in front than behind); and propodeum smooth with very few discrete large punctures except very short transverse carina each side along the median keel and dense rugulosity around spiracles (the latter with propodeum coarsely rugose).

### Diolcogaster praritas

Zeng & Chen sp. n.

urn:lsid:zoobank.org:act:CD345F6F-2076-4B67-9A1F-EC1323648030

http://species-id.net/wiki/Diolcogaster_praritas

Figs 57–64

#### Description.

 Female. Body length 3.2 mm, fore wing length 3.5 mm.

Head. Oval in anterior view, with antennal sockets high above middle level of eyes; transverse in dorsal view, 1.6× as wide as long and slightly narrower than mesoscutum, with sparse and short setae except the sharply constricted and highly polished area behind lateral ocelli. Ocelli of moderate size, in a low, very wide triangle, the transverse, posterior tangent to the anterior ocellus just cutting the posterior pair. POL:OD:OOL=6.0:3.4:7.0. Vertex and frons smooth and shiny, only between the ocelli and the eye-margin very sparsely punctate; vertex strongly convex; face slightly convex, feebly and transversely striate-punctate, without any trace of median keel, width of face 0.9× as high as eye and clypeus combined (20.0:23.2); eyes small, inner margin of eyes adjacent to face parallel, 1.6× as high as wide (20.5:13.0); temple and gena feebly rugose-punctate and shiny, densely setose. Clypeus slightly swollen, feebly rugose; tentorial pits of moderate size, distance between tentorial pits 2.6× as long as distance from pit to eye margin (10.4:4.0); malar space short, 0.3× as long as eye height. Antennae a little shorter than the body, with the preapical segment about 1.5× longer than wide; scape short; flagellomeres long, thick, without bristly setosity, with placodes arranged regularly in 2 ranks except the distal 6 or 7 segments; apical segment slightly longer than preapical one. Flagellomere proportion: 2 L/W=3.6, 8 L/W=2.5, 14 L/W=2.1; L 2/14=1.4; W 2/14=0.8.

Mesosoma. Pronotum with a very indistinct ventral furrow laterally. Mesoscutum with dense rugose-puctures but interspaces shiny, with short setae all over; notauli not impressed, but indicated by a band of broad depression posteriorly. Disc of scutellum finely, discretely punctate, very shiny, with normal setosity, polished at tip, so the posterior, polished band of scutellum continuous. Lateral part of the polished band at least distinctly convex anteriorly. Anterior margin of the postscutellum closely applied to the posterior margin of the scutellum so that, laterally, the phragma of scutellum is completely hidden. Scutellar sulcus shallow, with few longitudinal carinae, its width almost as long as scutellum (15.0:15.5). Propodeum short, shiny, finely reticulate-rugose all over, with a well-defined median keel. Mesopleuron in large part polished. Precoxal sulcus very short, indicated by a shallow depression with dense but shallow punctures.

Wings. Forewing with rather large 4-sided areolet; vein r(1st abscissa of the radius) much longer than 3-SR(2nd abscissa of the radius), arising from distal 1/2 of pterostigma, far behind the middle, placed at right angles to the pterostigma, meeting vein 2-SR at a 150 degree angle. Vein 1-R1 (metacap) with distal end sharply defined, 3.5× as long as its distance from the apex of the marginal cell and 1.2× as long as pterostigma; pterostigma 2.7× as long as wide; r:2-SR:length of pterostigma=9.0:10.0:31.0. Width of 1st discal cell:height of 1st discal cell =26.5:23.0, 1-CU1:2-CU1:m-cu=9.0:13.6:10.0. Hind wing with vannal lobe convex, fringed with long setae throughout.

Legs. Long and thin. Hind coxa very feebly punctate, almost polished. Hind tibia swollen apically and about 0.8× as long as hind tarsus (55.0:68.2), outer surface without distinct spines. Inner hind tibial spur much longer than outer one, 0.6× as long as hind basitarsus (17.0:30.0); fourth tarsal segment shorter than fifth tarsal segment (7.2:10.0); apical segment of the front tarsus without a spine Tarsal claws simple.

Metasoma. T1 almost parallel-sided, with a shallow median groove over basal 2/3 that with ill-defined sides; dull and coarsely rugose, with long setae laterally and sparse short setae dorsally, 1.4× as long as its width and 1.5× as long as T2. T2 trapezoidal, with a shiny and feebly longitudinally striated raised median field, irregularly shaped but distinctly narrowed behind; the lateral fields striate-rugose, but sculptures reduce toward lateral margin, 1.5× times as wide as long and 1.1× as long as T3. Tergites posterior to T2 highly polished and smooth, more membranous, with short fine setae posterolaterally; setae of metasoma very sparse, almost absent on T3. Ovipositor sheath with 2–3 blackened setae at apex. Hypopygium large, evenly sclerotised, smooth and shiny, with fine setae subapically, with apex a little surpassing the last tergite.

Colour. Body almost black, metasoma yellow on anterior half ventrally and brown dorsally. Antennae light brown at base and darkened toward apex to brown. Clypeus brownish, mouthparts brownish yellow, with ventral margin of mandible brown; palpi whitish yellow. Tegula light brown. Legs almost virtually yellow, except hind coxa brown at extreme base and hind tibia and tarsuli slightly brownish. Tergites dark brown, except lateral margin of T2 and posterolateral corner of T3 yellowish. Tergites posterior to T2 somewhat transparent. Wings hyaline; veins and pterostigma brown, somewhat transparent.

Variation. Paratype distinctly smaller than holotype; T3 yellowish on posterior half and pterostigma broader.

**Figures 57–64.**
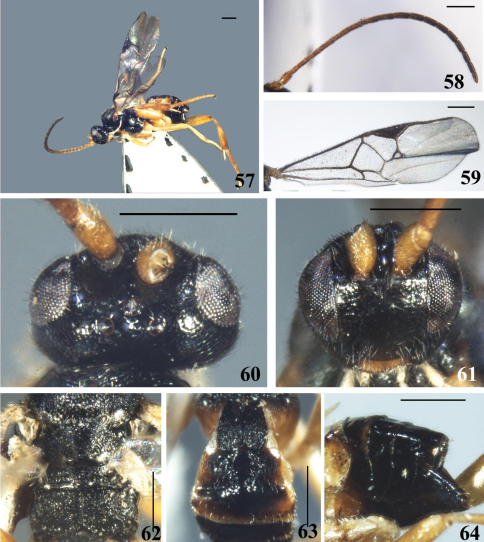
*Diolcogaster praritas* Zeng & Chen, sp. n. **57** habitus, lateral view **58** antennae **59** fore wing **60** head, dorsal view **61** head, frontal view **62** scutellum and propodeum, dorsal view **63** T1–3, dorsal view **64** hypopygium and ovipositor sheaths, lateral view. Scale line = 0.5 mm.

#### Male.

 Unknown.

#### Host.

 Unknown.

#### Material examined.

 Holotype: ♀, Tongbiguan (97°38'E, 24°37'N), Yingjiang, Yunnan, 2009. V. 17, legs. Zeng Jie, No. 200904237. Paratype: 1♀, Mt. Fengyang (119°12'E, 27°55'N ~ 119°14'E, 27°56'N), Longquan, Zhejiang, 2007. VII. 28, legs. Liu Jingxian, No. 200802854.

#### Etymology.

 The specific name “*praritas*” derives from the Latin noun “praritas”, referring to the irregular median field of T2.

#### Distribution.

 China (Zhejiang, Yunnan).

#### Remarks.

 This species is similar to *Diolcogaster austrina* (Wilkinsonellus, 1929), but can be distinguished by the disc of scutellum finely, discretely punctate (the latter with disc of scutellum smooth and highly polished); pterostigma shorter than 1-R1 vein (the latter with pterostigma just longer than 1-R1 vein); and TII with surface of the lateral fields besides median field striate-rugose, but reduced toward lateral margin (the latter with the lateral fields besides median field unsculptured).

### Diolcogaster punctatiscutum

Zeng & Chen sp. n.

urn:lsid:zoobank.org:act:F470CE60-B6AF-406A-955C-59EE33E1E73E

http://species-id.net/wiki/Diolcogaster_punctatiscutum

Figs 65–72

#### Description.

 Female Body length 2.8 mm, fore wing length 3.2 mm.

Head. Oval in anterior view, with antennal sockets high above middle level of eyes; transverse in dorsal view, 1.6× as wide as long and almost as long as mesoscutum, with dense short fine setae except the sharply constricted and highly polished area behind lateral ocelli. Ocelli of moderate size, in a low, very wide triangle, the transverse, posterior tangent to the anterior ocellus just cutting the posterior pair. POL:OD:OOL=5.8:4.2:7.5. Vertex vey shiny, with very sparsely discrete punctures, strongly convex; frons polished, virtually without sculpture; face slightly convex, also very shiny, finely but densely punctate, with denser setae, without any trace of median keel, width of face 1.1× as high as eye and clypeus combined (26.8:24.1); eyes small, inner margin of eyes slightly widened downwards, 1.6× as high as wide (22.0:14.0); temple and gena shiny with denser and deeper punctures than vertex, and with longer setae. Clypeus indistinct, slightly convex and as finely and densely punctate as face; tentorial pits large, distance between tentorial pits 3.0× as long as distance from pit to eye margin; malar space short, 0.2× as long as eye height. Antennae longer than body, with the preapical segment fully twice as long as wide; scape short; flagellomeres thinner, tappered distally, without bristly setosity, with placodes arranged regularly in 2 ranks; apical segment longer than preapical one. Flagellomere proportion: 2 L/W=2.5 , 8 L/W=3.1, 14 L/W=2.0; L 2/14=2.1; W 2/14=1.0.

Mesosoma. Pronotum shiny, with a very broad foveate ventral furrow laterally. Mesoscutum very shiny, evenly and densely punctate, with short fine setae all over; notauli not impressed. Disc of scutellum with denser punctures, larger laterally, with interspaces shiny, very densely setose; polished at tip, so the posterior, polished band of scutellum is continuous. Lateral part of the polished band distinctly convex anteriorly. Anterior margin of the postscutellum closely applied to the posterior margin of the scutellum so that, laterally, the phragma of scutellum is completely hidden. Scutellar sulcus deep and broad, with few longitudinal carinae, its width as long as scutellum (15.0:15.0). Propodeum short, shiny, reticulate-rugose all over, coarsely rugose laterally and with strong rugae around spiracles, with a strong complete median keel. Mesopleuron in large part polished (including area below precoxal sulcus posteriorly), the other part with setigerous-punctures. Precoxal sulcus only indicated on anterior half of mesopleuron, shallow and broad, feebly and largely foveate anteriorly and polished behind.

Wings. Forewing with areolet 4-sided; vein r (1st abscissa of the radius) much longer than 3-SR (2nd abscissa of the radius), arising from distal 1/2 of pterostigma, slightly behind the middle, placed at almost right angle to the pterostigma, meeting vein 2-SR at a 160 degree angle. Vein 1-R1 (metacarp) with distal end sharply defined, 3.9× as long as its distance from the apex of the marginal cell and as long as pterostigma; pterostigma 3.1× as long as wide; r:2-SR:length of pterostigma=12.5:5.0:32.5. Width of 1st discal cell:height of 1st discal cell =24.3:24.0; 1-CU1:2-CU1:m-cu=6.0:14.0:10.0. Hind wing with edge of vannal lobe beyond its widest part straight and without trace of a fringe of setae.

Legs. More or less stout. Hind coxa very shiny, with fine and very sparse punctures and dense short fine setae, inner surface with much shorter setae; rather small, not reaching to T3. Hind tibia swollen apically and 1.1× as long as hind tarsus (49.0:46.3), outer surface with darkened but very indistinct spines. Inner hind tibial spur longer than outer one, 0.8× as long as hind basitarsus (13.0:17.0); fourth tarsal segment much shorter than fifth tarsal segment (5.4:8.9); apical segment of the front tarsus without a spine. Tarsal claws simple.

Metasoma. Broad. T1 parallel-sided or slightly swollen medially, with anterior half surface smooth and shiny and sharply separated from coarsely and strongly reticulate-rugose posterior half surface by a strong carina on each side of median groove; the percurrent median groove indistinct on anterior half and sharply rimmed on posterior half, with few long setae in the middle laterally, 1.1× as long as its width, and almost twice as long as T2; T2 transverse, shortened medially, with a raised pentagonal median field, shiny but densely rugulose all over; 2.8× times as wide as long and 1.2× as long as T3. T3 transverse with anterior margin curved, shiny with very fine rugulosity and a few short fine setae. Tergites posterior to T3 more membranous, highly polished with a row of short fine setae bordering anterior margins except for the last two segments which only have sparse short fine setae. Ovipositor sheath with even width, with a fine modified apical seta. Hypopygium large, evenly sclerotised, smooth and shiny, with long fine setae, apex slightly surpassing the last tergite.

Colour. Body dark brown to black, except metasoma yellow with brown apex. Antennae light brown at base and darkened toward apex to brown. Mouthparts lighter with mandible yellow and palpi white. Tegula whitish yellow. Legs almost virtually yellow, except apical 1/3 of hind tibia and apex of claws brown and hind basitarsus slightly brownish. Tergites almost yellow and somewhat transparent, except middle of T3 to T6 and hypopygium, T7, posterior half of T8, the last tergite and ovipositor sheaths brown. Wings hyaline; veins brown, pterostigma dark.

**Figures 65–72.**
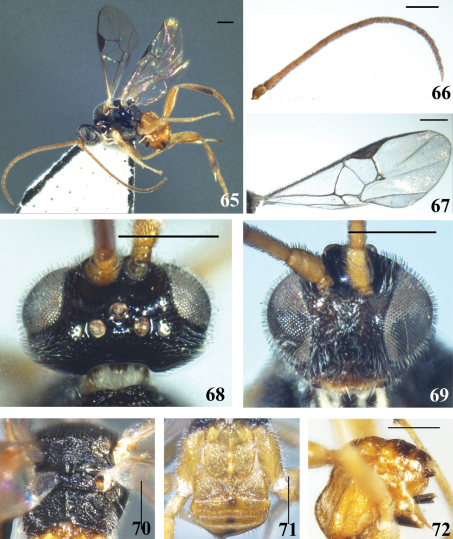
*Diolcogaster punctatiscutum* Zeng & Chen, sp. n. **65** habitus, lateral view **66** antennae **67** fore wing **68** head, dorsal view **69** head, frontal view **70** scutellum and propodeum, dorsal view **71** T1–3, dorsal view **72** hypopygium and ovipositor sheaths, lateral view. Scale line = 0.5 mm.

#### Male.

 Unknown.

#### Host.

 Unknown.

#### Material examined.

 Holotype: ♀, Fengxi (116°15'E, 24°31'N ~ 116°17'E, 24°32'N), Meizhou, Guangdong, 2003. VII. 29, legs. Chen Jujian, No. 20048449.

#### Etymology.

 The specific name “punctatiscutum” derives from the Latin adjective “punctate” and noun “scutum”, referring to disc of scutellum with large punctures.

#### Distribution.

 China (Guangdong).

#### Remarks.

 This species is similar to *Diolcogaster austrina* (Wilkinsonellus, 1929), but can be distinguished by the disc of scutellum with dense punctures, larger laterally (the latter with disc of scutellum smooth and highly polished); vein 1-CU1 much shorter than vein 2-CU1 (the latter with vein 1-CU1 and vein 2-CU1 of equal length); and antennae normal, long and thin (the latter with antennae short and stout).

### Diolcogaster spreta

(Marshall, 1885)

http://species-id.net/wiki/Diolcogaster_spreta

Microgaster spreta Marshall 1885: 259. Holotype female, United Kingdom. Marshall 1890: 547; Fahringer 1937: 329; Telenga 1955: 195.
Protomicroplitis spreta : Nixon 1965: 255; You et al. 1990: 47.

#### Host.

*Dioryctria palumbella*; *Euzophera consociella*.

#### Distribution.

 China (Shaaxi); Czechoslovakia, Hungary, Moldova, United Kingdom.

#### Remarks.

 No specimens were available for this study.

### Diolcogaster translucida

Zeng & Chen sp. n.

urn:lsid:zoobank.org:act:468601AA-83BF-4DCD-995E-4C091996C016

http://species-id.net/wiki/Diolcogaster_translucida

Figs 73–80

#### Description.

 Female. Body length 3.0 mm, fore wing length 3.4 mm.

Head. Rather large, oval in anterior view, with antennal sockets high above middle level of eyes; strongly transverse in dorsal view, 1.4× as wide as long and 0.8× as long as that of mesonotum, with short white setae including eyes. Ocelli in a low, very wide triangle, the transverse, posterior tangent to the anterior ocellus just cutting the posterior pair. POL:OD:OOL=7.1:3.8:8.0. Vertex densely rugose, strongly convex and sharply constricted to occiput, with very short dense fine setae; frons also densely rugose; face slightly convex, rugose and setose, upper half with very indistinct median longitudinal carina, width of face 0.8× as high as eye and clypeus combined (15.0:27.2); eyes of moderate size, inner margins of eyes slightly narrowed downwards, 1.8× as high as wide (27.2:15.0); temple and gena feebly rugose-punctate and shiny, densely setose. Clypeus also rugose and setose; tentorial pits small, distance between tentorial pits 2.8× as long as distance from pit to eye margin (12.8:4.6); malar space short, 0.2× as long as eye height. Antennae slightly longer than body, with the preapical segment about thrice as long as wide; flagellomeres with bristly setosity, with placodes arranged regularly in 2 ranks except the apical one; apical segment subequal to preapical one in length. Flagellomere proportion: 2 L/W=2.9, 8 L/W=2.5, 14 L/W=2.3; L 2/14=1.6; W 2/14=1.3.

Mesosoma. Pronotum with two weakly indicated but distinct foveate furrows laterally. Mesoscutum shiny, evenly and densely rugose-punctate, with short setae all over; notauli not impressed. Disc of scutellum as strongly sculptured as the mesoscutum, with normal setosity, its rugose tip at middle widely interrupting the posterior, polished band of scutellum. Lateral, polished field reduced to a thin, parallel-sided strip. Anterior margin of the postscutellum closely applied to the posterior margin of the scutellum so that, laterally, the phragma of scutellum is completely hidden. Scutellar sulcus deep, with few longitudinal carinae, its width 0.9× as long as scutellum (19.0:20.4). Propodeum shiny, with a complete median keel; surface on each side of the median propodeal keel almost smooth, with dense obsolescent punctures, only with very short transverse ridging on immediate vicinity of longitudinal carina. Mesopleuron polished posteriorly and above precoxal sulcus, depressed below and there densely setigerous-punctate; precoxal sulcus long, broad, shallow with shallow longitudinal carina.

Wings. Forewing with vein r-m reduced to a mere hyaline point and more or less interstitial with vein r so that the areolet is virtually 3-sided, very small, slit-like. Vein r arising from distal 1/2 of pterostigma, far behind the middle, very obliquely placed on the pterostigma, meeting vein 2-SR at a 100 degree angle. Vein 1-R1 (metacarp) with distal end almost reaching to apex of marginal cell, 2.4× as long as pterostigma, pterostigma 2.1× as long as wide; r:2-SR:length of pterostigma=14.0:9.5:21.0. Second discoidal cell setose almost everywhere. Width of 1st discal cell:height of 1st discal cell =23.0:24.5. 1-CU1:2-CU1:m-cu=12.0:10.2:10. Hind wing broad, with vannal lobe beyond its widest part straight and fringed with short setae throughout.

Legs. Long and thin. Hind coxa large, just reaching past posterior margin of T3, with evenly and closely punctate surface, the interspaces very shiny. Hind tibia swollen toward apex and about 0.9× as long as hind tarsus (57.0:66.2), with rather sparse fine spines. Inner hind tibial spurs much longer than outer ones, about 0.8× as long as hind basitarsus (24.0:30.0); fourth tarsal segment shorter than fifth tarsal segment(9.2:10.0); apical segment of the front tarsus without a spine. Tarsal claws rather long, simple.

Metasoma. Slightly shorter than mesosoma. Tergites with short fine setae all over. T1 almost parallel-sided, roundly constricted at apical 1/5, with complete longitudinal groove; smooth, except for rugosity at posterior corners, 1.4× as long as its width, 1.6× as long as T2. T2 strongly transverse, anterior margin oblique each side besides the middle, with a more or less distinct parallel-sided median field that elongated and smooth, the lateral fields are not transverse and more or less aciculate-rugose, 0.5× as long as its width and 1.4× as long as T3. T3 also transverse, membranous, polished, separated from T2 by a deep groove. Tergites posterior to T3 more membranous; setae of metasoma very sparse, almost absent on T3. Ovipositor sheath with 2–3 strong and blackened modified setae at apex. Hypopygium small, evenly sclerotised, smooth with sparse fine setae, not surpassing the last tergite.

Colour. Body black, more or less brownish, except that metasoma yellow to brown. Antennae light brown, slightly darkened toward apex. Mouthpart yellow, palpi paler. Tegula whitish yellow. Fore and mid legs uniformly bright yellow; hind coxa black but brownish marginally, trochanter yellow, femur brown with the extreme base yellow; hind tibia lighter than femur but on basal half bright yellow, hind spurs bright yellow. T1 bright yellow, somewhat transparent, T2 and T3 brown; other tergites light brown. Wings hyaline; veins and pterostigma light brown, transparent.

Variation. Some individuals with head and hind coxa yellow.

**Figures 73–80.**
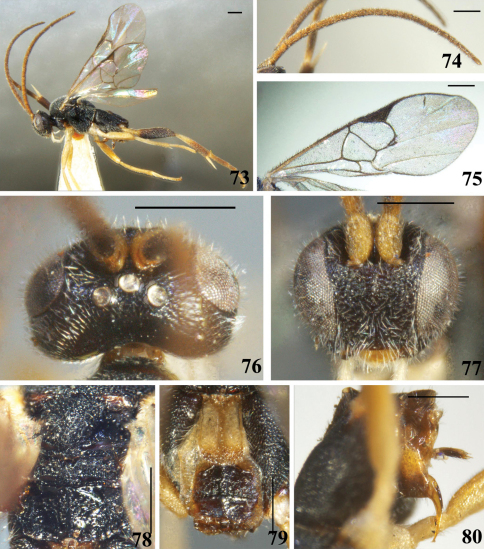
*Diolcogaster translucida* Zeng & Chen, sp. n. **73** habitus, lateral view **74** antennae **75** fore wing **76** head, dorsal view **77** head, frontal view **78** scutellum and propodeum, dorsal view **79** T1–3, dorsal view **80** hypopygium and ovipositor sheaths, lateral view. Scale line = 0.5 mm.

**Figures 81–88.**
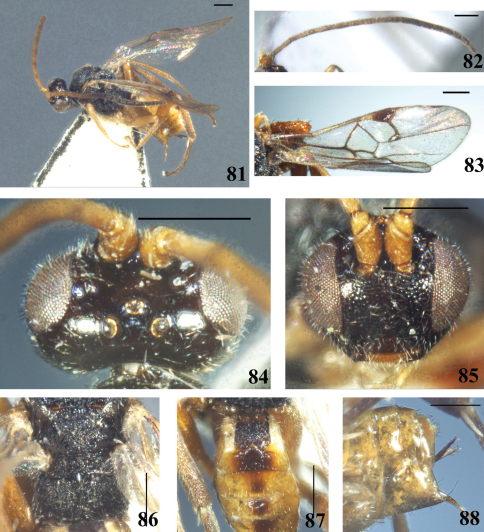
*Diolcogaster perniciosa*(Wilkinson, 1929) **81** habitus, lateral view **82** antennae **83** fore wing **84** head, dorsal view **85** head, frontal view **86** scutellum and propodeum, dorsal view **87** T1–3, dorsal view **88** hypopygium and ovipositor sheaths, lateral view. Scale line = 0.5 mm.

#### Male.

 Unknown.

#### Host.

 Unknown.

#### Material examined.

 Holotype: ♀, Yiliping (117°40'E, 27°43'N ~ 117°42'E, 27°44'N), Fujian, 1981. V. 5, legs. Huang Juchang, No. 20004177. Paratype: 1♀, Tianbaoyan, Yongan (117°17'E, 25°54'N ~ 117°27'E, 26°02'N), Fujian, 2001. VII. 15–18, legs. Xu Zaifu, No. 20020245; 1♀, Mt. Longqi, Jiangle (117°11'E, 26°23'N ~ 117°21'E, 26°43'N), Fujian, 1991. VII. 16, legs. Liu Changming, No. 20007159; 1♀, Mt. Tongledashan (111°20'E, 23°07'N ~ 111°29'E, 23°14'N), Yunan, Guangdong, 2003. VIII. 12–13, legs. Xu Zaifu, No. 20054526; 1♀, Fengxi (116°15'E, 24°31'N ~ 116°17'E, 24°32'N), Meizhou, Guangdong, 2003. VII. 29, legs. Chen Jujian, No. 20048755; 1♀, Mt. Nanling (112°59'E, 24°53'N ~ 113°05'E, 24°56'N), Ruyuan, Guangdong, 2003. VII. 23, legs. Xu Zaifu, No. 20049030; 1♀, Mt. Gutian (118°07'E, 29°14'N ~ 118°10'E, 29°16'N), Zhengjiang, 2005. V. 2, legs. Wu Qiong, No. 200616757; 1♀, West Mt. Tianmu (119°23'E, 30°20'N ~ 119°24'E, 30°20'N), Linan, Zhejiang, 1998. V. 25, legs. Zhao Mingshui, No. 20003376; 3♀♀, Baotianman (111°55'E, 33°29'N ~ 111°58'E, 33°32'N), Neixiang, Henan, 1998. VII. 14, legs. Chen Xuexin, No. 988345, 988654, 988701; 1♀, Mt. Huping (110°45'E, 30°02'N ~ 110°55'E, 30°07'N), Shimen, Hunan, 2009. VII. 9, Zeng Jie, No. 200901332.

#### Etymology.

 The specific name “*translucida*” derives from the Latin adjective “translucidus”, referring to the colour of the whole body more or less transparent.

#### Distribution.

 China (Henan, Zhejiang, Fujian, Hunan, Guangdong).

#### Remarks.

 This species is similar to *Diolcogaster xanthaspis* (Ashmead, 1900), but can be distinguished by the ovipositor sheath with a modified apical seta (the latter without); and propodeum with surface on each side of the median propodeal keel smooth-liking, its sculpture reduced to coarse, obsolescent punctuation (the latter with propodeum coarsely rugose).

## Supplementary Material

XML Treatment for Diolcogaster

XML Treatment for Diolcogaster alvearia

XML Treatment for Diolcogaster bifurcifossa

XML Treatment for Diolcogaster brevivena

XML Treatment for Diolcogaster chaoi

XML Treatment for Diolcogaster grammata

XML Treatment for Diolcogaster ineminens

XML Treatment for Diolcogaster laetimedia

XML Treatment for Diolcogaster perniciosa

XML Treatment for Diolcogaster pluriminitida

XML Treatment for Diolcogaster praritas

XML Treatment for Diolcogaster punctatiscutum

XML Treatment for Diolcogaster spreta

XML Treatment for Diolcogaster translucida
